# Prospective Associations between Maternal and Child Diet Quality and Sedentary Behaviors

**DOI:** 10.3390/nu13051713

**Published:** 2021-05-18

**Authors:** Charlotte Juton, Carles Lerin, Clara Homs, Rafael Casas Esteve, Paula Berruezo, Gabriela Cárdenas-Fuentes, Montserrat Fíto, Maria Grau, Lidia Estrada, Santiago F. Gómez, Helmut Schröder

**Affiliations:** 1 Endocrinology Department, Institut de Recerca Sant Joan de Déu, 08950 Barcelona, Spain; charlottejuton@gmail.com (C.J.); clerin@fsjd.org (C.L.); 2 PhD Program Food and Nutrition University of Barcelona, 08028 Barcelona, Spain; 3 Gasol Foundation, 08830 Barcelona, Spain; choms@gasolfoundation.org (C.H.); pberruezo@gasolfoundation.org (P.B.); sgomez@gasolfoundation.org (S.F.G.); 4 PSITIC Research Group, Psychology, Education and Sport Sciences Department, Blanquerna–Universitat Ramon Llull, 08022 Barcelona, Spain; 5 CSM Nou Barris, Programa de Jóvenes, 08042 Barcelona, Spain; rafael.casas@csm9b.com; 6 Non-Communicable Diseases and Environment Research Program, ISGlobal Barcelona Institute for Global Health, 08003 Barcelona, Spain; gabriela-cardenas@isglobal.org; 7 Cardiovascular Risk and Nutrition Research Group, IMIM Hospital del Mar Medical Research Institut, 08003 Barcelona, Spain; mfito@imim.es; 8 CIBER Physiopathology of Obesity and Nutrition (CIBERobn), Instituto de Salud Carlos III, 28029 Madrid, Spain; 9 Cardiovascular Epidemiology and Genetics Research Group, IMIM Hospital del Mar Medical Research Institut, 08003 Barcelona, Spain; mgrau@imim.es; 10 CIBER Epidemiology and Public Health (CIBERESP), Instituto de Salud Carlos III, 28029 Madrid, Spain; 11 Serra Hunter Fellow, Departament of Medicine, University of Barcelona, 08007 Barcelona, Spain; 12 Gasol Foundation, Los Angeles, CA 90004, USA; lestrada@gasolfoundation.org; 13 GREpS, Health Education Research Group, Nursing and Physiotherapy Department, University of Lleida, 25008 Lleida, Spain

**Keywords:** mothers, children, diet quality, sedentary behaviors, prospective associations

## Abstract

As the most likely primary caregivers, mothers are an integral part of children’s social influence and are therefore greatly involved in shaping their children’s behaviors. The objectives were to determine the prospective associations between maternal and child diet quality and sedentary behaviors. This study, within the framework of a community-based intervention study, included 1130 children aged 8–10 years and their mothers. The study was carried out during two academic years (2012/2014) with a mean follow-up of 15 months. Exposure and outcome variables were measured at baseline and follow-up, respectively. Diet quality was assessed by the KIDMED questionnaire and the short Diet Quality Screener, respectively. Sedentary behaviors were determined by standardized questions of sedentary behaviors. Maternal consumption of fruits, vegetables, fish, legumes, pasta/rice, dairy products, nuts and baked goods were positively associated (*p* < 0.05) with the corresponding child behavior. Multiple linear regression models adjusted for sex, age, maternal education and intervention group revealed significant cross-sectional (*p* < 0.005) and prospective (*p* < 0.01) associations between maternal and child overall diet quality and sedentary behaviors. Maternal diet quality and sedentary behaviors were predictive for these lifestyle behaviors in children.

## 1. Introduction

Poor dietary habits during childhood may hinder cognitive development and inhibit school achievement [1]. Alongside sedentary behaviors, they can also lead to excess weight gain [2,3] and poor health outcomes including cardiovascular disease and pre-diabetes [4]. Therefore, the early introduction of healthy lifestyle behaviors can have a positive impact on children’s health [3] and wellness [5]. Healthy lifestyle habits comprise, among others, intake of nutrient-rich foods [4] especially at breakfast [1] and limiting sedentary behaviors [4].

In Spain, the Childhood Obesity Surveillance Initiative (COSI) 2015-17 reported that children aged 6 to 9 years old rarely skip breakfast [6]. However, the main foods consumed for breakfast were milk, baked goods and cereals [7]. Furthermore, the consumption of fruits and vegetables was low in that population [7], whereas children almost never consumed savory snacks and sugary soft drinks [6,7]. With regard to sedentary behaviors, a substantial proportion of children reported watching television or using electronic devices at least 2 h per day on weekdays and weekends [6,7].

Numerous cross sectional [8,9,10,11,12,13,14,15,16,17,18,19] and few longitudinal [20,21,22] studies have shown that parents may influence their children dietary and sedentary habits. In relation to children’s dietary habits, a meta-analysis [9] demonstrated a weak to moderate correlation with those of the parents and a longitudinal study showed that from all lifestyle mothers diet displayed the greatest positive correlation [22]. In a cross-sectional study, Spanish children whom parents had healthier eating attitudes were less likely to present micronutrients inadequacy and marginally more likely to adhere to a Mediterranean diet [18]. With respect to sedentary behaviors, a prospective study indicated that mothers watching TV daily presented a higher risk of having children watching TV an hour or more [21] and two clustered cross-sectional studies showed that mothers’ TV time were either negatively associated with children meeting screening recommendations [17] or positively associated with TV time ≥ 2 h per day [16]. A recent cross-sectional study also found a positive correlation between Brazilian mothers or fathers’ and adolescents’ sedentary behaviors [19]. Despite large cross-sectional evidence, longitudinal results are still lacking therefore this study aimed to determine the prospective associations between the habits of mothers and their children with respect to dietary quality and sedentary behaviors.

## 2. Materials and Methods

### 2.1. Study Design

Data were taken from the POIBC prospective study which stands for Prevention of Childhood Obesity: a Community-based Model in Spanish. The entire POIBC protocol has been published elsewhere [23]. In brief, the POIBC intervention study assessed the efficacy of the THAO-Child Health Program in order to prevent childhood obesity in 2249 children aged 8 to 10 years [24]. The study was carried out during two academic years (2012/2014) with a mean follow-up of 15 months. Data from intervention and control groups were integrated to study the longitudinal associations. All the participants variables, from intervention and control cities, were gathered at baseline and at average follow-up. After excluding missing data on any of the included variables, a total of 1130 participants (570 boys and 560 girls) remained; therefore, the prospective cohort for this study included 1130 mother-child dyads. The local ethics committee (CEIC-PSMAR, Barcelona, Spain, approval number: (2011/4296/I) approved the study. Children were informed about the study and about the fact that their participation is voluntary. Written consent was obtained on behalf by their parents.

### 2.2. Maternal Diet Quality

Mothers’ dietary quality was recorded at home through the self-administered short Diet Quality Screener (sDQS) [25]. This questionnaire captured usual dietary behaviors over the previous 12 months by assessing three levels of habitual intake of 18 food items in three food categories. The first category included eight items: (i) bread, (ii) vegetables/salads, (iii) fruit, (iv) yoghurt or milk, (v) pasta or rice, (vi) olive oil, (vii) alcoholic beverages and (viii) breakfast flakes. The second category contained seven items: (i) meat, (ii) sausages, (iii) cheese, (iv) pastry or sweets, (v) butter or lard, (vi) other vegetable oils and (vii) fast food. Finally, the third category contained three items: (i) fish, (ii) legumes and (iii) nuts. Excluding alcoholic beverage consumption, daily intake of one portion of foods in the first food group category accounted for 1; lower and higher intakes accounted for 0 and 2, respectively. Daily consumption of one alcoholic drink (one bottle of beer, one glass of wine, or one glass of liquor equivalent to approximately 12g of alcohol) accounted for 2; lower and higher intakes accounted for 0. Consumption of foods deemed pernicious in the second food group category accounted for 1 if reported as 4–6 times per week; more and less frequent consumption accounted for 0 and 2, respectively. High consumption (4 or more times per week) of food items deemed favorable of the third food group category accounted for 2. Intakes of 2–3 times and less than twice a week accounted for 1 and 0, respectively. All food items scores were summed up, giving a total score ranging from 0 (low-quality diet) to 36 (high-quality diet).

### 2.3. Maternal Sedentary Behaviors

Mothers were asked to indicate how much time they spent in sedentary behaviors (television viewing, computer work, and playing videogames during the week and on weekends based on the self-administered REGICOR short physical activity questionnaire [26]. Total time per day was computed as follows: [(sedentary time weekdays × 5) + (sedentary time weekend × 2)]/7 days.

### 2.4. Child Adherence to the Mediterranean Diet

Children’s Mediterranean diet adherence was examined using the KIDMED index [27]. The KIDMED questionnaire was administered in schools with the assistance of trained field researchers at baseline and at follow-up. The KIDMED questionnaire includes 16-items and estimates Mediterranean diet adherence in children and young adults based on the dietary principles of the Mediterranean diet. This index follows a binary response format (yes/no). Four items indicating lower adhesion were assigned a value of − 1 [Goes more than once a week to a fast-food restaurant; skips breakfast; has commercially baked goods or pastries for breakfast; takes sweets and candy several times every day] and the 12 items related to greater adherence were rated + 1 [takes a (serving/piece of) fruit or fruit juice every day; has a second (serving/piece of) fruit every day; has fresh or cooked vegetables once a day; has fresh or cooked vegetables more than once a day; consumes fish regularly; likes legumes and eats them more than once a week; consumes pasta or rice almost every day (five or more times per week); has cereals or grains (bread, etc.) for breakfast; consumes nuts regularly (at least 2–3 times per week); uses olive oil at home; has a dairy product for breakfast (yoghurt, milk, etc.); consumes two yoghurts and/or some cheese (40 g) daily]. The range of scores was between − 4 and 12, with higher scores reflecting greater adherence to the Mediterranean diet. Adherence was classified as low (≤3 points), medium (4–7 points) or high (≥ 8 points). The KIDMED questionnaire did not ask for food consumption/habits in a previous timeframe.

### 2.5. Comparison between Maternal and Child Eating Behaviors

Maternal food consumption was recorded at home by the sDQS and compared with the corresponding food items of the KIDMED questionnaire. In brief, maternal food consumption of each food item was dichotomized as follows: category (i) fruits, vegetables, pasta/rice, cereals/grains at breakfast, olive oil, and dairy products, consumed less than one time per day vs. one or more times per day; category (ii) fish, legumes, and nuts, consumed less than two times per week vs. two or more times per week; and (iii) fast food and baked goods, consumed fewer than four times per week vs. four or more times per week;. Categories were compared with the following corresponding items of the KIDMED questionnaire: (i) takes a (serving/piece of) fruit or fruit juice every day; has fresh or cooked vegetables once a day; consumes pasta or rice almost every day (five or more times per week); has cereals or grains (bread, etc.) for breakfast; uses olive oil at home; has a dairy product for breakfast (yoghurt, milk, etc.); (ii) consumes fish regularly; likes legumes and eats them more than once a week; consumes nuts regularly (at least 2–3 times per week) and (iii) goes more than once a week to a fast-food restaurant; has commercially baked goods or pastries for breakfast.

### 2.6. Child Sedentary Behaviors

Television viewing was used as a proxy for sedentary behaviors. Children indicated the average time they spent watching television during the week and on weekends based on the Screen time based Sedentary Behaviour Questionnaire (SSBQ) [28]. Total time per day was computed as follows: [(TV week × 5) + (TV weekend × 2)]/7 days.

### 2.7. Maternal Socioeconomic Status

Maternal education level was collected as an indicator of socioeconomic status and categorized into five levels: (i) no schooling, (ii) primary school, (iii) secondary school, (iv) technical or other university degree, and (v) higher (graduate-level) university degree.

### 2.8. Statistical Analysis

Cross-sectional (Model 1) and prospective analysis (Model 2) were performed. Logistic regression was performed to assess the baseline and prospective associations between the selected eating habits (dichotomous 0 and 1 all variables) for mothers and children. Model 1 was adjusted for sex (dichotomous, boy = 1 and girl = 2), age (baseline, continuous), intervention group (dichotomous, intervention group = 1 and control group = 2) and maternal education (baseline, dichotomous, less than university = 0 and university = 1). Model 2 was additionally adjusted for child’s corresponding food consumption at baseline.

Linear regression analysis was performed to determine the cross-sectional (Model 1) and prospective (Model 2) associations of maternal diet quality and sedentary behaviors with the corresponding child behavior. All models were adjusted for sex (dichotomous, boy = 1 and girl = 2), age (baseline, continuous), intervention group (dichotomous, intervention group = 1 and control group = 2) and maternal education (baseline, dichotomous, less than university = 0 and university = 1). Model 2 was additionally adjusted for child’s corresponding lifestyle (eating habits or sedentary behaviors) at baseline.

Linear regression models with cubic spline functions (gam package R) were fitted to analyze the dose-response relationship between maternal and child lifestyle behaviors. This model compares the likelihood between the model that assumes that the effect is linear with the more general model that admits that the effect may be nonlinear. Furthermore, the gam additive models estimate the effect of a quantitative trait or variable over the outcome which is supposed to follow a normal distribution and the possibly to control for other variables. In opposite to linear models, additive models do not suppose or impose a linear effect and can therefore be of any kind (quadratic, cubic, logarithmic, J-shape, etc.) with the only restriction to be smoothed. The models were adjusted for sex (dichotomous, boy = 1 and girl = 2), age (baseline, continuous), intervention group (dichotomous, intervention group = 1 and control group = 2), and maternal education (baseline, dichotomous, less than university = 0 and university = 1) and the corresponding child behavior at baseline.

The SPSS for Windows version 18 (SPSS, Inc., Chicago, IL, USA) was used for all statistical analysis except for the dose-response analysis which was performed using R Statistical Software (version 2.14.0; R Foundation for Statistical Computing, Vienna, Austria).

## 3. Results

Children who were excluded from analysis were somewhat older (10.16 ± 0.63 years) than those included (10.09 ± 0.60 years, p difference between groups = 0.008). There was no significant (*p* = 0.178) difference for gender between groups.

Table 1 presents the general characteristics of the participants. Table 2 shows descriptive data on the proportion of mothers and children with positive responses of questions on food consumption. The cross-sectional logistic regression analysis showed, except for fast food, that frequent consumption by children of fruits, vegetables, fish, legumes, pasta/rice, cereals/grains at breakfast, nuts, olive oil, dairy products and baked goods were positively associated with maternal intakes of the corresponding food groups after adjusting for age, sex, intervention group and maternal education (Table 3 Model 1).

The prospective logistic regression analysis was significant for child intake of fish, legumes, pasta/rice, nuts and baked goods and the matched maternal food group after adjusting for age, sex, intervention group and maternal education (Table 3 Model 2).

Maternal diet quality and sedentary behaviors were positively associated with the correspondent child lifestyle variables in the cross-sectional and prospective linear regression models adjusted for age, sex, intervention group and maternal education (Table 4).

**Figure 1:**
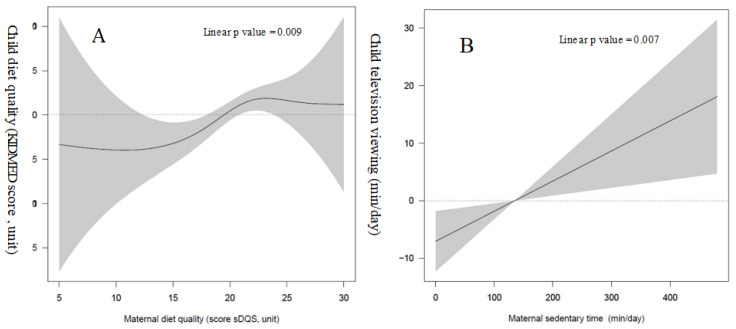
Prospective dose-response relationship of maternal diet quality (**A**) and sedentary behaviors (**B**) with the corresponding child lifestyle behaviors. Models were adjusted for age, sex, intervention group, maternal education, and the respective lifestyle behavior at baseline.

Linear regression models with cubic spline functions did not reveal a significant non-linear dose-response relationship between maternal and child diet quality and sedentary behaviors (Figure 1). Tests of interaction revealed no significant interaction between sex and adherence to the Mediterranean or sedentary behaviors. Sex stratified analysis showed similar directions and magnitudes of the associations in each stratum.

## 4. Discussion

In this study, mothers’ healthy eating habits, including the consumption of fish, legumes, nuts and pasta/rice, were prospectively associated with the comparable habit in children, which was also true for the consumption of baked goods. Furthermore, maternal overall diet quality and sedentary behaviors were positive predictors of their children’s corresponding lifestyle behavior after adjusting for age, sex, intervention group, maternal education.

Likewise, a published meta-analysis [9] found a weak to moderate correlation between parental and child dietary regimens. Across studies, dietary intake and dietary quality score between mothers and children were positively associated. However, a Dutch study [29] of maternal influence on children’s eating behavior found lower associations for food eaten outside the home compared to food eaten at home. These findings could be explained by the important influence that mothers have during early childhood as role models in shaping children’s behaviors within the shared home environment, especially healthy behaviors [30]. On the other hand, as a result of an increased awareness of child obesity, mothers may limit access to unhealthy food inside the household or discourage poor health habits like the consumption of junk food outside the home [30].

A systematic review and meta-analysis [31] supported the importance of role modelling and availability of healthy vs. unhealthy foods within the home environment. Restrictive guidance towards food consumption yielded mixed results, with some studies showing a decrease and others an increase in the consumption of unhealthy food items [31]. Similarly, a representative US study [32] found that modelling and availability of fruits, vegetables, fast food consumption and sugar-sweetened beverages within the household were associated with a higher consumption of fruits and vegetables as well as lower consumption of fast food and sugar-sweetened beverages. However, it stressed that setting rules and limits on fast food and sugar drinks was associated with an increased consumption of those products among adolescents. Parental disapproval of eating junk food may trigger a desirability effect, leading adolescents who tend to spend less time at home and more time with their friends [33] to fulfil a perceived need outside the home environment, where the maternal influence is less strong; this increases the risk of obesity [34].

Similar to our study, an American prospective study [21] reported maternal TV exposure as a predictor of infants’ television viewing. Additionally, two representative clustered cross-sectional studies, carried out in Australia and Greece, found that maternal television viewing time was positively associated with television viewing time by their young children (aged 3 to 5 years) and negatively associated with their children meeting screen-time recommendations [16,17]. A Canadian study in children aged 0 to 5 years also reported a higher risk of increased screen-time when parents reported a screen-time of at least 43 min per day [35]. Other parental associated factors were of particular interest to acknowledge the interdependence between parents and children’s sedentary behaviors. Factors positively associated with sedentary behaviors included: weather, safety, parental house chores, having a television in the child’s room, parental perception of normal daily screen-time [12] and infant crying duration [21]. Conversely negative factors encompassed parental education, income, capacity to say no to screen-time [12] and parental or friend support to physical activity [36].

The dose-response analysis showed a significant linear association between maternal and child sedentary behaviors and diet quality. This indicates that both maternal behaviors linearly improve the corresponding child behavior.

The key strengths of this study are its prospective design and relatively large sample size. The main study limitation is the use of self-reported questionnaires, which are vulnerable to inherent measurement errors. A further methodological limitation is the fact that questions on food consumption were recorded by different questionnaires among children and their mothers. Therefore, frames of the sDQS and KIDMED questionnaire are somewhat different. However, this is an inherent limitation when comparing dietary data retrieved by different short diet screeners or food frequency questionnaires. Another limitation is that TV watching was used as a proxy for sedentary behaviors in children. However, in epidemiological studies TV viewing seems to be an acceptable proxy to sedentary behaviors [37] when the use of accelerometers, also subject to measurement errors by their lack of distinction between sitting and standing, cannot easily be administered.

In this study several mothers’ healthy eating habits were prospectively associated with the comparable habit in children. Additionally, after adjusting for age, sex, intervention group and maternal education, maternal overall diet quality and sedentary behaviors were positive predictors of children’s corresponding lifestyle behaviors.

## 5. Conclusions

Our study demonstrated that maternal eating habits and sedentary behaviors were related to those of their children in this sense designing interventions that reinforce maternal healthy habits cannot only be beneficial to mothers but also to children. The earliest healthy habits are introduced, the more likely they are to last over time, therefore further research might want to explore mothers’-children’s diet and sedentary behaviors associations at an early age until the adolescence period to acknowledge how maternal behaviors condition those of their children on the long term.

## Figures and Tables

**Table 1:** General characteristics of the study population (*n* = 1130).

	Baseline	Follow-Up ^6^
Girls % (n)	49.6 (560)	49.6 (560)
Age (years)	10.1 ± 0.6	11.3 ± 0.6
Maternal education ^1^ % (n)	35.6 (402)	-
Child diet quality ^2^ (unit)	6.9 ± 2.4	6.6 ± 2.4
Maternal diet quality ^3^ (unit)	19.9 ± 3.7	20.8 ± 3.2
Child sedentary behaviors ^4^ (min/d)	56.7 ± 49.1	66.7 ± 50.2
Maternal sedentary behaviors ^5^ (min/d)	135.1 ± 73.4	135.2 ± 85.2

Continuous and categorical variables are expressed as mean (standard deviation) and proportion (n) of participants, respectively. ^1^ More than primary education ^2^ KIDMED index ranges from −4 to 12. ^3^ sDQS score ranges from 0 to 36. ^4^ Child television viewing (min per day). ^5^ Maternal sedentary behaviors (television viewing, computer work, and playing videogames, min per day). ^6^ Maternal diet quality *n* = 626; maternal sedentary behavior *n* = 779.

**Table 2:** Percentage of mothers’ and children’s positive response for the food items (*n* = 1130).

Food Consumption	Mothers (Baseline, %)	Children (Baseline, %)	Children (Follow-Up %)
Fruit ^1^	81.2	68.6	67.6
Vegetables ^1^	80.0	59.2	58.7
Pasta or rice ^1^	41.8	51.0	48.3
Cereals ^1^	28.3	69.5	68.4
Olive oil ^1^	90.9	90.1	92.6
Dairy products ^1^	89.2	85.6	87.0
Fish ^2^	58.4	66.7	69.1
Legumes ^2^	48.3	64.4	66.8
Nuts ^2^	28.0	45.8	42.3
Fast-food ^3^	13.5	18.1	28.8
Baked goods or pastries ^3^	17.0	21.4	19.5

^1^ Maternal consumption of fruits, vegetables, pasta or rice, cereals, olive oil and dairy products ≥ 1 time/day. ^2^ Maternal consumption of fish, legumes and nuts ≥ 2 times/week. ^3^ Maternal consumption of fast-food and baked good.

**Table 3:** Baseline and prospective associations between maternal and child food consumption.

	Fruit ^1^	Vegetables ^1^	Pasta/Rice ^1^	Cereals/Grains at Breakfast ^1^	Olive Oil ^1^	Dairy Products ^1^
	OR 95% CI	OR 95% CI	OR 95% CI	OR 95% CI	OR 95% CI	OR 95% CI
**Model 1 ^2^**						
<1 time/day	Reference	Reference	Reference	Reference	Reference	Reference
≥1 time/day	1.69 1.24; 2.30	1.89 1.40; 2.54	1.33 1.04; 1.69	1.63 1.21; 2.20	2.35 1.37; 4.03	1.82 1.22; 2.74
*p*-value	**0.001**	**<0.0001**	**0.021**	**0.001**	**0.002**	**0.004**
**Model 2 ^3^**						
<1 time/day	Reference	Reference	Reference	Reference	Reference	Reference
≥1 time/day	1.28 0.93; 1.78	1.34 0.98; 1.83	1.56 1.22; 2.00	1.18 0.87; 1.60	0.98 0.46; 2.08	1.37 0.87; 2.17
*p* value	0.14	0.77	**<0.0001**	0.29	0.95	0.18
	**Fish ^1^**	**Legumes ^1^**	**Nuts ^1^**			
	OR 95% CI	OR 95% CI	OR 95% CI			
**Model 1 ^2^**						
<2 times/week	Reference	Reference	Reference			
≥2 times/week	2.90 2.24; 3.74	1.65 1.28; 2.11	1.84 1.41; 2.40			
*p* value	**<0.0001**	**<0.0001**	**<0.0001**			
**Model 2 ^3^**						
<2 times/week	Reference	Reference	Reference			
≥2 times/week	1.631.23; 2.17	1.60 1.23; 2.08	1.54 1.17; 2.03			
*p*-value	**0.001**	**<0.0001**	**0.002**			
	**Fast food ^1^**	**Baked goods ^1^**				
	OR 95% CI	OR 95% CI				
**Model 1 ^2^**						
<4 times/week	Reference	Reference				
≥4 times/ week	0.83 0.53; 1.32	1.36 0.94; 1.96				
*p* value	0.44	0.10				
**Model 2 ^3^**						
<4 times/week	Reference	Reference				
≥4 times/week	0.97 0.65; 1.43	1.50 1.02; 2.22				
*p*-value	0.87	**0.041**				

^1^ Multiple logistic regression analysis was used to determine the associations between child and maternal diet comparable items. Values are expressed as odds ratio (OR) and 95% confidence intervals 95% CI. ^2^ Model 1: cross-sectional association adjusted for sex (dichotomous, boy = 1 and girl = 2), age baseline, continuous, intervention group (dichotomousintervention group = 1 and control group = 2), and maternal education baseline, (dichotomous (less than university = 0 and university = 1). ^3^ Model 2: prospective association adjusted for adjusted for sex (dichotomous, boy = 1 and girl = 2); age baseline, continuous, intervention group (dichotomous, intervention group = 1 and control group = 2); maternal education baseline (dichotomous, less than university = 0 and university = 1), and for child’s corresponding food consumption at baseline.

**Table 4:** Baseline and prospective associations between child and maternal diet quality and sedentary behaviors.

	β	95% CI	*p*-Value
Diet quality (score unit) ^1^			
Model 1 ^2^	0.122	0.085–0.159	<0.0001
Model 2 ^3^	0.045	0.011–0.079	0.009
Sedentary behaviors (min/d) ^1^			
Model 1 ^2^	0.058	0.019–0.970	0.004
Model 2 ^3^	0.052	0.014–0.090	0.007

^1^ Linear regression analysis was used to determine the associations between maternal (exposure) and child diet quality and sedentary behaviors (outcome). Values are expressed as β coefficients and 95% confidence intervals (95% CI). ^2^ Model 1: cross-sectional association adjusted for sex (dichotomous, boy = 1 and girl = 2), age baseline (continuous), intervention group (dichotomous, intervention group = 1 and control group = 2) and maternal education baseline (dichotomous, less than university = 0 and university = 1). ^3^ Model 2: prospective association adjusted for adjusted for sex (dichotomous, boy = 1 and girl = 2), age baseline, continuous, intervention group (dichotomous, intervention group = 1 and control group = 2) and maternal education baseline (dichotomous, less than university = 0 and university = 1).and baseline values of the corresponding dependent variable.

## Data Availability

Data and materials are available upon request to the corresponding authors.
